# From Hand to Eye With the Devil In-Between: Which Cognitive Mechanisms Underpin the Benefit From Handwriting Training When Learning Visual Graphs?

**DOI:** 10.3389/fpsyg.2021.736507

**Published:** 2021-10-27

**Authors:** Tânia Fernandes, Susana Araújo

**Affiliations:** Faculdade de Psicologia, Universidade de Lisboa, Lisbon, Portugal

**Keywords:** sensorimotor training, handwriting, letter processing, reading, visual analysis

## Abstract

Cognitive science has recently shown a renewed interest on the benefit from training in handwriting (HW) when learning visual graphs, given that this learning experience improves more subsequent visual graph recognition than other forms of training. However, the underlying cognitive mechanism of this HW benefit has been elusive. Building on the 50 years of research on this topic, the present work outlines a theoretical approach to study this mechanism, specifying testable hypotheses that will allow distinguishing between confronting perspectives, i.e., symbolic accounts that hold that perceptual learning and visual analysis underpin the benefit from HW training vs. embodied sensorimotor accounts that argue for motoric representations as inner part of orthographic representations acquired *via* HW training. From the evidence critically revisited, we concluded that symbolic accounts are parsimonious and could better explain the benefit from HW training when learning visual graphs. The future challenge will be to put at test the detailed predictions presented here, so that the devil has no longer room in this equation.

## Introduction

Literacy is an exquisite example of human ingenuity. Written scripts are composed by *graphs*^1^, i.e., artificial two-dimensional geometric-like shapes (cf. Chang et al., 2018) that are arbitrary but, when learning to read, become visual counterparts of linguistic units as phonemes, syllables, or morphemes (e.g., letters in the Latin alphabet, kanas and kanjis in Japanese), and gears of written words (e.g., Pelli et al., 2003; Grainger, 2018). Reading thus bridges visual object recognition and language.

Along with reading development, a hierarchically organized, orthographically tuned circuitry is built along the *visual ventral stream*, originally dedicated to visual recognition of familiar objects (for a review, see Dehaene et al., 2015). Skillful graph and visual word recognition depend on fast access to abstract orthographic representations (usually called *abstract or symbolic letter identities*) which are not determined by physical (visual, low-level), phonological, or motor similarity and are underpinned by the left *ventral occipitotemporal* cortex, *vOT* (Dehaene et al., 2005; Rothlein and Rapp, 2014). Readers’ immunity to physical differences of *allographs* (visual forms of the same graph) is found in different scripts: e.g., in Latin alphabet, A and a; in Japanese kanas, 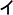 and 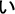; in Arabic abjad, 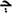 and 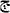 (e.g., Bowers, 1996; Carreiras et al., 2013; Kinoshita et al., 2019). It depends on long-term changes in the perceptual space of graphs, which are consequence of learning to read. For example, B is physically equidistant to both p and b, and hence, without any experience, observers would be as fast in discriminating the pair B-p as the pair B-b. However, readers are slower in discriminating the pair B-b in consonance with the degree of perceptual similarity of graph representations (Lupyan et al., 2010; for recent evidence with training on an artificial script, see, Wiley and Rapp, 2021). This example demonstrates that learning to read is an ecological example of perceptual learning (Gibson, 1969, 1970; Dehaene et al., 2005; Goldstone and Byrge, 2015).

Probably because learning to read puts heavy demands on visual processing, the observation that motor training *via handwriting, HW* (that is, writing by hand) benefits more subsequent visual graph recognition than other learning experiences has been startling since the earliest studies (Jeffrey, 1958; Williams, 1969, 1975; Jensen and King, 1970; Koenigsberg, 1973). The advantage from HW training when learning visual graphs is robust. It is found in different written systems and types of scripts (for a recent meta-analysis, see Araújo et al., 2021; e.g., Jensen and King, 1970; Guan et al., 2011; Cao et al., 2013b; Li and James, 2016; Xu et al., 2020; Vinci-Booher et al., 2021). It is especially strong for *highly confusable* graphs which share visual features and in the most extreme the whole shape, differing only by orientation, such as *mirror images* (e.g., d and b) or rotations in the image plane (i.e., *plane rotation*: e.g., N and Z, or n and u) (Hendrickson and Muehl, 1962; Williams, 1969, 1975; Torres et al., 2020). It is found relative to *visual-only* (e.g., looking at) and to *motor control* (e.g., typewriting on a keyboard, pointing to, circling) training, regardless of training in phonological correspondences, adoption of natural or artificial scripts, amount of training (single vs. multiple training sessions), and age of learners (Araújo et al., 2021; for an overview, see James, 2017; e.g., Williams, 1969; Longcamp et al., 2006, 2008; James, 2010; Bara and Gentaz, 2011; Guan et al., 2011; Suggate et al., 2016; Labat et al., 2020; Mayer et al., 2020; Seyll et al., 2020). Note that control training often leads to improvement in subsequent visual graph recognition as well, but HW training usually excels it (Longcamp et al., 2006; Kiefer et al., 2015; Labat et al., 2020; Mayer et al., 2020; Fernández-López et al., 2021). This benefit from HW is impressive because it corresponds to a *transfer* effect (Gilbert and Li, 2012): performance in an untrained (non-motor) visual recognition task on the new graphs is enhanced *via* HW training, indicating that neural plasticity is not restricted to the brain underpinnings of the graphomotor task but extends to those of visual graph recognition at the left fusiform gyrus in the vOT (Longcamp et al., 2008; James, 2010).

It is consensual that writing by hand, stroke by stroke, establishes a connection between the visual percept of the graph and the motor plan for creating it, resulting in a sensorimotor experience that influences learning to read. Most research has provided descriptive insight only, occasionally legitimating evidence-based programs (e.g., Bara et al., 2016; Mayer et al., 2020; Torres et al., 2020) given that HW is a worldwide strategy in literacy instruction (e.g., Tan et al., 2005; Bara and Gentaz, 2011; Itaguchi et al., 2015; Xu et al., 2020). However, this benefit from HW training does not necessarily imply that knowledge on how to write the graphs (that is, on motoric representations) is the underlying mechanism. This (premature) conclusion confuses the expression of a learning experience with the underlying cognitive representations and processes (Norris and Cutler, 2021).

In fact, explanatory insights have been rare (notwithstanding recent exceptions: Li and James, 2016; Zhai and Fischer-Baum, 2019; Seyll et al., 2020; Vinci-Booher et al., 2021; Wiley and Rapp, 2021). We thus came to have misgivings about the underlying mechanism: Why does HW training benefit visual graph recognition? Is it due to perceptual learning and visual segmental analysis regardless of the motor act (e.g., Williams, 1975; Courrieu and de Falco, 1989; Seyll et al., 2020)? Or does it depend on internal motor simulation (e.g., Longcamp et al., 2008, 2016) such as that of stroke order during visual graph recognition (e.g., Parkinson and Khurana, 2007; Itaguchi et al., 2015)?

It is time to put the phenomenon into perspective, confronting potential explanations, in order to bring to light the underlying cognitive mechanism. In the present work, we critically review evidence from pioneering research (e.g., Hendrickson and Muehl, 1962; Pick, 1965; Williams, 1969; Jensen and King, 1970; Koenigsberg, 1973) to more recent functional resonance imaging (fMRI) studies (e.g., Longcamp et al., 2006; James and Atwood, 2009; James, 2010; Cao et al., 2013b; Vinci-Booher et al., 2021). Our aim is to aggregate this research, adopting a theoretically based perspective that has hitherto been largely missing. Specifically, we consider the two alternative theoretical frameworks (about conceptual representations, and hence, not exclusive of letter representations) that can be associated with most research on the benefit from HW training even if this theoretical discussion has often been dismissed (e.g., Bara and Gentaz, 2011; Labat et al., 2020; Torres et al., 2020). The debate between these two theoretical perspectives is about the *nature* of cognitive representations, that is, about their *format* (i.e., the nature of the code used to represent information) and not about their content (that is, which information is stored; for a critical discussion, see, e.g., Glenberg et al., 2013; Barsalou, 2016; Goldinger et al., 2016; Machery, 2016; Mahon and Hickok, 2016).

On the one hand, *embodied* cognitive accounts (also called grounded or situated cognition: e.g., Allport, 1985; Barsalou, 2008; Glenberg et al., 2013) hold that the content and the format of cognitive representations is isomorphic: sensory concepts have a sensorial format and action concepts have a motor format. Therefore, representations of graphs and of written words would not be symbolic but rather *sensorimotor*. During visual graph recognition, the graphic motor programs acquired *via* HW would be *reactivated* or *simulated* because they would be an inner part of graph representations. This sensorimotor mechanism would be responsible for the benefit from HW training in visual graph recognition (e.g., Bara and Gentaz, 2011; Longcamp et al., 2016; Labat et al., 2020; Xu et al., 2020). Action-perception coupling *via* HW training would be critical for the development of the reading-specialized circuitry (Longcamp et al., 2008; James and Atwood, 2009) because reading would involve a gesture decoding system, located within a region of the left *dorsal premotor cortex, PMd* (Brodmann Area 6; e.g., Longcamp et al., 2008; Nakamura et al., 2012; Cao et al., 2013b).

On the other hand, *abstractionist, symbolic* accounts (e.g., Goldinger et al., 2016; Machery, 2016; Mahon and Hickok, 2016) do not deny the role of sensorimotor experiences when learning to read but hold that abstract representations are not reducible to, and hence differ from, modality-dependent ones. In what regards visual graph processing, ample behavioral, neuroimaging, and neuropsychological evidence (e.g., Rapp and Caramazza, 1997; Dehaene et al., 2005; Lupyan et al., 2010; Dufor and Rapp, 2013; Wiley and Rapp, 2021) shows that symbolic, amodal graph identities are core representations in reading and writing in alphabetic and non-alphabetic scripts (e.g., Carreiras et al., 2013; Rothlein and Rapp, 2014; Kinoshita et al., 2019). These abstract orthographic representations are connected *via* bidirectional links to the input (visual) and output (motor) systems, with automatic spreading of activation between them (Rapp and Caramazza, 1997; Dufor and Rapp, 2013). HW training would thus assist on the emergence of abstract orthographic representations due to activation dynamics (that is, because activation cascades automatically) and not because motoric representations were an inner part of orthographic representations. In this vein, HW training would benefit subsequent visual graph recognition due to the operation of a *perceptual learning* mechanism, resulting in long-lasting changes in the perception of the trained graphs (e.g., Gibson, 1969, 1970; Williams, 1975; Courrieu and de Falco, 1989; Goldstone and Byrge, 2015; Seyll et al., 2020).

Both frameworks predict that HW training benefits more subsequent visual graph recognition than other learning experiences. However, they disagree about the nature of the representations and about the putative mechanisms underpinning the HW benefit. In this work, we highlight some of the loose ends that research has left and present our theoretical framework and hypotheses. Before our proposal, we start by pinpointing the cognitive components involved in HW, to then discuss three promising theoretical accounts framed by the symbolic cognitive framework (i.e., the perceptual variability and the visual analysis hypotheses) and by the embodied cognitive framework (i.e., the stroke processing hypothesis). For each hypothesis, we first present critical positive evidence and next the evidence that questions it. Note, however, that it is not our aim to provide an exhaustive literature review but one that is unbiased and tackles the critical evidence for the present discussion. We also detail the predictions that follow, because, like the devil, unveiling the underlying cognitive mechanisms is on the details.

## Why Is Handwriting So Special? Three Theoretical Proposals Looking for the Common Denominators

Learning to read is often accompanied by proxies of HW as copying and tracing (Kiefer et al., 2015; Mayer et al., 2020). It is a multisensory experience bridging visual graphs with speech sounds and motor gestures (Pegado et al., 2014). Consequently, letters have multiple codes (i.e., visual, motor, phonological) and are involved both in reading and in writing, which in turn also comprise multiple types of representations (Abbott and Berninger, 1993; Rothlein and Rapp, 2014). Therefore, the cognitive mechanisms and representations involved in a transfer effect from HW training to visual graph recognition are not straightforward. It is undeniable that HW training has effects in writing and might also have in other abilities beyond the written domain (Abbott and Berninger, 1993). However, the scope of the present work is on the benefit from HW training on subsequent visual recognition of graphs. We are isolating a specific challenge that is posed when learning to read, that is, the emergence of abstract graph identities, which can be distinguished from other challenges posed in orthographic processing during reading development (e.g., transposed letter effect; phonological consistency).

The first strategy to enlighten the transfer effect from HW training to visual graph recognition is by process decomposition. The rationale here is that of the perceptual expertise literature (Curby and Gauthier, 2010): the training task is a vehicle for encouraging differences in processing and/or in representation of graphs, and transfer effects from training to testing tasks depend on the common denominators (Gilbert and Li, 2012). Such rationale is not only optimal to theory testing but can also readily translate into education. By identifying the key components of this benefit, other tasks besides HW can be used to optimize learning to read. Furthermore, given that HW is becoming an obsolete task in the digital era, its inclusion in school activities depends on the contribution of HW to other facets of literacy beyond writing *per se* (Wiley and Rapp, 2021). For unveiling this cognitive mechanism, we need to isolate the common denominators, that is, which representations and processes in the learning experience are critical to subsequent visual graph recognition and reading. To this aim, we first consider the cognitive components involved in HW.

Handwriting is a perceptual-motor multi-component task that involves a plethora of processes, including balance, eye-hand coordination, focused attention, visual processing, fine coordination of hand movements, and precise motor control of spatial and temporal constraints (Waterman et al., 2015; Julius et al., 2016). Behaviorally, HW is characterized by *legibility* and *fluency* (i.e., accuracy and speed in reproduction). It is underpinned by a frontoparietal associative striatum-cerebellar circuitry that also engages attention and executive processes (Makino et al., 2016; Palmis et al., 2017; Vinci-Booher et al., 2019). Therefore, at first sight, multiple possible mechanisms could be involved in a transfer effect to visual graph recognition and reading (for a similar discussion, see, Wiley and Rapp, 2021).

Notably, the temporal course of HW learning reveals two aspects that could be key for unveiling the underlying cognitive mechanism. Like other forms of motor learning, HW follows a well-characterized temporal course of two phases, both dependent on the *cortical-cerebellar* loop (the primary motor, somatosensory, dorsal premotor, and parietal cortices, the thalamus and cerebellum) and the *cortico-striatal* loop (composed by the same motor cortical areas, the thalamus and basal ganglia; Makino et al., 2016; Palmis et al., 2017). It begins with a *fast-learning phase* of rapid improvement but with slow and highly variable graph reproduction. Movements are overly guided by the visual stimuli, with exploration of multiple motor behaviors, contingently to the resulting visual output (Maldarelli et al., 2015). Next, in a *slow-learning* phase, refinement of graph reproduction occurs over a longer time course. HW becomes legible, fluent, and highly stereotyped but still relies more on visual feedback than proprioceptive (tactile and kinesthetic) feedback (Palmis et al., 2017). Thus, the two key aspects are that, during the first phase, HW is accompanied by highly variable visual outputs, and hence, perceptual variability of the graphs to-be-learned is maximum, and in both learning phases visual processing is prevalent.

This temporal course also highlights the first loose end of this topic of research. Most studies on the benefit from HW training when learning visual graphs likely tap mostly into the first phase of motor learning. These studies were usually short-term, many with a single training session of 1–3 min (e.g., Naka and Naoi, 1995; Suggate et al., 2016) to 20–25 min (e.g., Jensen and King, 1970; Guan and Wang, 2017) and subsequent visual graph recognition was tested immediately or 24 h post-training (e.g., Williams, 1969; James and Atwood, 2009). From the 50 studies meta-analyzed by Araújo et al. (2021), half had just one or two training sessions (of less than 30 min) and only six studies had more than seven training sessions (e.g., Kiefer et al., 2015; Mayer et al., 2020). Follow-up testing has been rare, although post-training gains are kept after at least one week (Longcamp et al., 2006, 2008; Cao et al., 2013b; Seyll et al., 2020; Vinci-Booher et al., 2021). Note, however, that when children were trained on real letters, uncontrolled post-training exposure could have been involved in the follow-up results (e.g., Longcamp et al., 2005). More important to the present work, in these training studies HW is always preceded, accompanied, and followed by visual graph processing, and hence, visual perceptual processes seem to be critical. This observation agrees with fMRI evidence showing enhancement of functional activity in occipitotemporal regions for HW with ink vs. without ink (Vinci-Booher et al., 2019). Therefore, in short-term training studies, HW training is contingent to visual pattern learning, and hence, its benefit could be due to perceptual learning of graphs (Williams, 1975; Courrieu and de Falco, 1989).

The first two hypotheses discussed here, that is, the *perceptual variability* (James and Engelhardt, 2012; Li and James, 2016) and the *visual analysis* (Koenigsberg, 1973; Courrieu and de Falco, 1989; Seyll et al., 2020) hypotheses are framed by symbolic accounts of cognitive representations (e.g., Goldinger et al., 2016; Machery, 2016; Mahon and Hickok, 2016). Both were originally proposed in studies with preliterate children, and hence, with learners that had no previous reading expertise in any script (we return to this point in Section “The nature of graph representations”). These hypotheses are not mutually exclusive but focus on different operations of perceptual learning. As aforementioned, this mechanism is responsible for the emergence of symbolic graph identities (Gibson, 1969; Dehaene et al., 2005; Goldinger et al., 2016) as expressed by faster discrimination of the pair B-p than B-b by Latin-alphabet readers (Lupyan et al., 2010). In fact, this example illustrates the two perceptual challenges that a learner faces, each one emphasized by one of the two symbolic hypotheses discussed below.

### The Perceptual Variability Hypothesis

First, *category learning* is used to abstract away over perceptual differences between allographs (e.g., B and b), giving rise to the formation of abstract letter identities at the left vOT (Dehaene et al., 2005; Rothlein and Rapp, 2014). These representations are the gateway for letter and visual word recognition across reading development (Grainger, 2018). Fast access to them is demonstrated by the observation of equivalent facilitation in recognition of written words preceded by identical items written in a different case, regardless of visual similarity of allographs: i.e., same magnitude of cross-case identity priming for e.g., <ROSE> preceded by as for <KISS> preceded by <kiss> (e.g., Bowers, 1996). It could thus be the case that, due to the high perceptual variability inherent to graph reproduction, HW training would assist on extraction of perceptual invariants (Kirk, 1980) relevant for the emergence of abstract letter representations. In other words, during HW, learners are exposed to variable (messy) visual stimuli more than in other types of training without a graphomotor activity (James and Engelhardt, 2012; Li and James, 2016). Thus, HW training could broaden graph categories at the left vOT due to *perceptual variability* (James and Engelhardt, 2012; Li and James, 2016): “experiencing visual variability would be more important for letter learning and subsequent visual recognition than experiencing the motor variability” (Vinci-Booher and James, 2020, p. 3).

Indeed, regardless of training including HW or not, children exposed to more variable instances of graphs show better reading abilities (Bara et al., 2016) and larger post-training gains in subsequent categorization of these graphs (Li and James, 2016). Nonetheless, when instances of new graphs are more variable by using a hampering writing tool (e.g., vibrating or conic-shape pen), learners show smaller post-training gains that those who used a regular pen (Suggate et al., 2016; Seyll and Content, 2020). However, a trade-off might have occurred between exposure to more variable instances of graphs and the attentional resources demanded by (difficult) motor reproduction with a hampering tool. In this regard, more conclusive evidence has been provided by Wiley and Rapp (2021). When adults were exposed to the same number of variable instances of Arabic letters, those trained *via* HW still showed faster learning rates of these letters than participants trained in visual or motor control conditions (Wiley and Rapp, 2021). Thus, albeit relevant, perceptual variability might not be the sole (or even the core) operation.

### The Visual Analysis Hypothesis

Second, learners become able to isolate diagnostic features *via differentiation*, leading to between-category expansion (e.g., B and p; Gibson et al., 1962). The most fascinating consequence of perceptual learning is that stimuli that were at first indistinguishable became discriminated, as happens with mirror images (e.g., b and d), which are originally processed as equivalent percepts due to *mirror-image invariance*. This property of the ventral visual stream is inherited from evolution: natural objects are often symmetric, and hence, for fast identification (i.e., whether an item is a tiger or a kitten) there is no advantage in discriminating mirror images which are just profile views of the same item (Bornstein et al., 1978; Logothetis et al., 1995; Pegado et al., 2014; Dehaene et al., 2015). However, mirror-image discrimination must be accomplished when learning a script with mirrored graphs as the Latin alphabet (e.g., p and q) or Japanese hiragana (e.g., 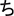 and 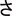) (Gibson et al., 1962; Kaufman, 1980; Kolinsky et al., 2011; Fernandes et al., 2016, 2021; for recent evidence on the role of the writing direction in mirror-image processing during lexical access, see, e.g., Soares et al., 2019, 2021).

The benefit from HW training could be because HW enhances awareness of the critical, distinctive features of graphs (Williams, 1969, 1975). *Visual features* are image components that are detected independently and are unaffected by the presence of other features; they are the primitives of visual object recognition (e.g., Pelli et al., 2003, 2006; Dehaene et al., 2005; Grainger, 2018). Therefore, the benefit from HW could be due to enhancement of visual-feature based processing, which in turn grounds efficient visual graph recognition (Gibson et al., 1962; Pelli et al., 2006; Grainger, 2018). This *visual analysis hypothesis* was the first proposed (for an early review, see, e.g., Kaufman, 1980), has recently seen a revival (Seyll et al., 2020), and is the one which gathers more corroborating evidence.

Handwriting training would foster discrimination of fine-grained visual configurations (Mayer et al., 2020). Therefore, as long as training engages explicit segmentation and visual discrimination, even if the graphomotor action is out of the equation, we would still get the same magnitude of gains in graph recognition. The empirical evidence available is coherent with this prediction. Indeed, explicit (non-motor) training on the distinctive features of highly confusable graphs is critical for facilitating graph recognition and subsequent letter-sound learning (Pick, 1965; Tawney, 1972; Samuels, 1973; Nelson and Wein, 1974). There is no added value of HW when visual training is fully focused on diagnostic features like orientation (e.g., d and b; Koenigsberg, 1973; Williams, 1975) nor when visually segmented graphs are presented to the learner (Courrieu and de Falco, 1989; Seyll et al., 2020). Visual exposure is not enough; training must imply visual analysis (e.g., Caldwell and Hall, 1969; Samuels, 1973; Spectorman et al., 1977).

Although appealing, most of the corroborating evidence is behavioral, and only a few studies have directly compared (non-motor) visual analysis training with HW training (Williams, 1969, 1975; Koenigsberg, 1973; Courrieu and de Falco, 1989; Seyll et al., 2020). Nonetheless, this theoretical account is also coherent with eye-movement patterns showing strong inspection of the visual item before copying it (Maldarelli et al., 2015) and transient enhancement at the left vOT for graphs learned *via* HW, immediately after training (James, 2010; James and Engelhardt, 2012; Vinci-Booher et al., 2021). Nonetheless, given that this hypothesis has not been directly tested in neuroimaging studies, it is still unknown whether such visual segmental training could lead to the same long-term neurocognitive changes in visual graph processing found after HW training, including those outside the vOT.

### The Stroke Processing Hypothesis

Notably, other mechanism could be involved. Along training, HW becomes automatic (legibility reaches a plateau by 2nd-grade, but HW only becomes automatic around the 3rd-grade, between 8 and 11 years of age; Waterman et al., 2015; Julius et al., 2016; Palmis et al., 2020). One generates similar graph shapes with different limbs and execution modes, suggesting that *abstract*, *effector-independent* motoric representations are involved. These representations specify graphs in terms of *strokes*, that is, units of movement defined by velocity vectors. Strokes can be segmented based on the occurrence of pen velocity minima, as happens, for example, when lifting off the pen because the beginning and end of the movement segment corresponds to an interruption (Rapp and Caramazza, 1997; Julius et al., 2016; Palmis et al., 2017). These representations are underpinned by the *PMd*, also known as a *graphemic motor image center* (see, e.g., Roux et al., 2009), whose damage often leads to *agraphia*, a specific writing disorder, with HW impairment (e.g., Kurosaki et al., 2016). This brain region is involved in transforming abstract motoric identities into motor plans (Dufor and Rapp, 2013). During HW by fluent readers, the PMd is specifically responsive to letter shape but not to letter identity (i.e., when shape changes; e.g., d and D). Naturally, HW training leads to the emergence of these motoric representations, which are necessary for legibility and fluency in subsequent writing tasks (Naka and Naoi, 1995; Kiefer et al., 2015; Wiley and Rapp, 2021). In this sense, the specificities of the sensorimotor learning experience are relevant for the emergence of motoric representations. However, it does not necessarily imply that these motoric representations are the ones responsible for the transfer effects from HW training to visual graph recognition. The point of dispute here regards the format of cognitive representations, on which embodied and symbolic cognitive accounts diverge.

The *stroke processing hypothesis* (e.g., Tan et al., 2005; Parkinson and Khurana, 2007; Itaguchi et al., 2015) was originally framed by an embodied cognitive account (e.g., Allport, 1985; Barsalou, 2008; Glenberg et al., 2013). According to it, the graphic motor programs acquired *via* HW would be reactivated during subsequent visual graph recognition because motor plans would be core of sensorimotor graph representations (Longcamp et al., 2008, 2016; James and Atwood, 2009; James and Gauthier, 2009; Labat et al., 2020). This hypothesis has been especially emphasized in logographic written systems because these graphs tend to be complex and with non-linear configurations (e.g., Chang et al., 2018), and hence, stroke simulation would be particularly relevant (Tan et al., 2005; Itaguchi et al., 2015): “writing facilitates recognition for both Chinese characters and English letters because (1) writing adds additional motor-related information to the representation system, which is wired together with visual input and enhances the activation of visual information during the recognition stage” (Cao et al., 2013b, p. 1671).

This hypothesis is appealing and has gathered considerable positive evidence, which we present next. However, it also has important limitations that have not hitherto been discussed in a systematic manner. Therefore, in the following Subsections “The nature of graph representations,” “Learners who are already experts in reading and HW,” and “Stroke processing is not about stroke order” we discuss the three caveats that question it.

Most enthusiasm with sensorimotor accounts and the stroke processing hypothesis has come from fMRI evidence during visual presentation of written stimuli. Such neuroimaging studies have found functional activity at the PMd and functional connectivity between frontal and/or parietal regions (within the writing network; e.g., Roux et al., 2009) and the vOT (part of the visual ventral stream and a core region of the reading network; e.g., Dehaene et al., 2005) in two populations: (i) In fluent adult readers presented with graphs or words written in the script on which they are experts (Longcamp et al., 2003; Nakamura et al., 2012; Vinci-Booher and James, 2020). (ii) In learners presented with visual graphs immediately after training (e.g., Longcamp et al., 2008; James and Atwood, 2009; James, 2010; James and Engelhardt, 2012; Vinci-Booher et al., 2016). This fMRI evidence has been interpreted as reflecting the involvement of motor representations during perception, because reading and writing would depend on sensorimotor representations of graphs (Longcamp et al., 2016; Vinci-Booher et al., 2016). Visual graph recognition would thus involve a gesture decoding system. Note, however, that fMRI evidence does not allow establishing causal inferences. We discuss this caveat in section “The nature of graph representations.”

Positive behavioral evidence was found with beginning readers. Indeed, copying of pseudographs by Chinese beginning readers was a reliable predictor of reading performance, even after controlling for general processing speed and phonological awareness (Tan et al., 2005). Adults learning Chinese as second language also showed larger post-training gains in hanzi categorization, not only after HW training, but also after training in an *animation* condition, where stroke order was presented unfolding but without a motor action involved (Xu et al., 2013). Furthermore, the post-training gains in graph naming by 2nd-grade Chinese children were similar (and larger than in control training) after HW training as after *kusho* training (air-writing training; Xu et al., 2020). Additionally, fluent adult readers of logographic scripts like Chinese or Japanese often adopt kusho and show better identification of decomposed kanjis when simultaneously doing kusho than when writing circles in the air or holding still (Itaguchi et al., 2015). However, this behavioral evidence regards participants that had already some prior (pre-training) knowledge of graphs. The effects of kusho training do not necessarily imply the involvement of motoric representations in visual graph recognition. These two aspects are discussed in section “Learners who are already experts in reading and HW.”

A *stroke order* effect has been reported in Latin-alphabet readers. When letters were presented as a sequence of strokes (dynamic unfolding, stroke-by-stroke), letter identification was faster in the consistent (left-to-right) than in inconsistent (right-to-left) stroke order (Parkinson and Khurana, 2007; Parkinson et al., 2010). Coherently, fluent readers show worse letter recognition when simultaneously writing another letter than when drawing a geometric shape. This *motor interference* by letters suggests that incongruent graphic motor programs were activated, interfering with visual graph recognition (James and Gauthier, 2009). In some patients with *alexia* (a specific reading disorder), HW also seems to facilitate letter recognition (e.g., Seki et al., 1995; Lott et al., 2010). The role of stroke processing was recently shown by Schubert et al. (2018) in Patient NGN (with a severe deficit in reading and in cross-case letter matching but with spared copying of letters and other symbols). When presented with letters comprising dots, Patient NGN showed worse letter identification for a static letter or a dynamic random one (not mimicking strokes) than for *dynamic* letters (dots presented in a continuous sequence along letter strokes) either in *consistent* or *inconsistent* orders. Note, however, that stroke processing is not the same as stroke order; the former is about the unit of movement and primitive of motor representations, while the latter is about the motoric program (the sequence of strokes) involved in graph reproduction. We return to this point in section “Stroke processing is not about stroke order.”

#### The Nature of Graph Representations

It is undeniable that sensorimotor accounts and the reviewed fMRI evidence are appealing. They are easy to understand and at first sight might seem *parsimonious*: they are brief, refer to observables, and have possible generality (Epstein, 1984; Vandekerckhove et al., 2015). However, fMRI evidence is correlational. It does not provide a causal explanation *per se* and neither does the mere reference to action-perception coupling due to brain-body-environment interaction (e.g., Longcamp et al., 2006; James and Atwood, 2009; Bara and Gentaz, 2011; Labat et al., 2020; Xu et al., 2020). To go beyond observation, it is necessary to bridge the evidence with psychological processes and mechanisms (Norris and Cutler, 2021).

Indeed, in at least some of the training studies on new graphs that have favored a sensorimotor account (e.g., Longcamp et al., 2005, 2006; James and Atwood, 2009; Bara and Gentaz, 2011), the benefit from HW training could be as easily explained by a perceptual learning hypothesis without reference to a sensorimotor mechanism, given that none of these studies has tested or discarded this alternative. Furthermore, even the most promising evidence for the stroke processing hypothesis, which has come from fMRI evidence of functional connectivity between visual and motor brain regions when learners were presented with graphs trained *via* HW (relative to control) immediately after training (e.g., Longcamp et al., 2008; James and Atwood, 2009; James, 2010; James and Engelhardt, 2012; Vinci-Booher et al., 2016) is limited. Indeed, Vinci-Booher et al. (2021) recently showed that such immediate enhancement in functional connectivity is temporary and not causally related with post-training gains in visual graph recognition, given that it was already gone (no functional connectivity observed) one week after training although the post-training gains in visual graph recognition were kept. These transitory effects are well accommodated by symbolic accounts considering the dynamics of activation spreading (Mahon and Hickok, 2016). Indeed, as aforementioned, ample evidence (Rapp and Caramazza, 1997; Dehaene et al., 2005; Lupyan et al., 2010; Dufor and Rapp, 2013; Rothlein and Rapp, 2014) shows that symbolic, amodal graph identities and sensorial and motor representations are independent but linked by bidirectional connections. Therefore, sensorimotor activity during letter perception would be about the dynamics of information flow rather than about the format of mental representations (Goldinger et al., 2016; Machery, 2016; Mahon and Hickok, 2016). In other words, visual graph recognition and graph production (writing) are related by means of abstract orthographic representations (e.g., Rapp and Caramazza, 1997; Rothlein and Rapp, 2014). Contrary to what some authors advocate (e.g., Longcamp et al., 2008, 2016; Cao et al., 2013a, b), when the evidence reported as favoring this hypothesis is thoroughly considered, it is unlikely that motoric representations and motor simulation during letter perception are responsible for the benefit from HW training in visual graph recognition.

Neuropsychological studies are also enlightening in this regard. Damage in brain regions responsible for HW does not necessarily lead to deficits in visual graph recognition and reading, even in Chinese (e.g., Bi et al., 2009; Kurosaki et al., 2016). Moreover, if graph representations were sensorimotor in format, then richer multisensory experiences would lead to larger post-training gains in graph recognition. However, the evidence says otherwise (Labat et al., 2020; Mayer et al., 2020; Xu et al., 2020; Araújo et al., 2021).

Critically, symbolic accounts have recently gained further support. A recent multi-session study demonstrated that HW training leads to the emergence of motoric representations but, importantly, also assists in the emergence of symbolic orthographic representations, which are dissociable from the former (Wiley and Rapp, 2021). In this study, three groups of participants learned Arabic letters along with their names and sounds. All were exposed for the same duration to multiple instances of dynamic letters (mimicking the stroke order) and to visually similar and visually dissimilar allographs: (e.g., 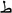 and 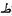; 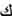 and 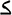, respectively), while performing an active task during training: (i) HW *via* copying; (ii) *typing* on a keyboard, where each allograph was presented on a specific key (hence, this motor control training comprised physically based discrimination including between allographs); (iii) *visual* by performing a same-different matching task (*different*-response trials corresponded to non-letter, familiar symbols, e.g., %, ?, #, and *same*-response trials to the letter being trained but in smaller size; hence, this visual training comprised a symbol/non-symbol categorization task). The most interesting result was found in a same-different matching task presented to participants at pre- and post-training. In this task, in *different*-response trials participants were presented with different graphs, which could be either different letters of Arabic (e.g., 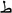 and 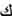) or allographs (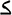 and 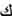). At post-training, the HW group showed sensitivity to motoric similarity but also to symbolic identity (i.e., slower *different*-response for allographs than for different graphs; cf. Lupyan et al., 2010). Furthermore, visual training also led to the emergence of symbolic representations in the absence of motoric ones.

#### Learners Who Are Already Experts in Reading and Handwriting

The second caveat stems from the fact that most positive evidence was found with participants who were not naïve on the graphs to be learned. In some studies, participants had already (at least some) knowledge of the script, given that they were beginning readers, either children (Tan et al., 2005; Xu et al., 2020) or adults learning Chinese as second language (who knew ∼180–450 hanzis, whereas fluent readers usually know ∼3000 hanzis; Cao et al., 2013a, b; Xu et al., 2013). Research with fluent adult readers (Parkinson and Khurana, 2007; James and Gauthier, 2009; Parkinson et al., 2010) or with alexic patients exposes the same caveat (Lott et al., 2010; Itaguchi et al., 2015; Schubert et al., 2018; Xu et al., 2020), given that such studies do not provide evidence on the mechanisms involved in learning the new graphs.

Research with kusho does neither provide conclusive evidence. Kusho or “air writing” corresponds to writing without visual feedback, and hence, to be successful in kusho, one needs to already know the visual form of graphs. In other words, for fluent readers kusho necessarily implies writing from memory; for learners, kusho training always occurs along with presentation of visual graphs (e.g., Itaguchi et al., 2015; Xu et al., 2020). In studies with fluent readers, the effects of kusho could thus be consequence of access to abstract letter identities (e.g., Itaguchi et al., 2015). Furthermore, the literate adults examined by Itaguchi et al. (2015) were asked to perform kusho while being presented with visually decomposed graphs, and hence, the effect could rather be about visual analysis. This alternative also applies to studies with learners. Indeed, Xu et al. (2020) found the same benefit in graph naming after HW training as after kusho training by the youngest group of 2nd-graders, and in both trainings, children were required to write the visual graph which was simultaneously presented on the screen. Therefore, the same motoric processes and the same explicit visual segmental analysis were operating in both conditions.

Pre-training knowledge of the graphs to-be-learnt might have a moderator role in the size of the benefit driven by HW. Specifically, post-training gains in visual graph recognition seem to be larger for children with less reading skills (Williams, 1969; Xu et al., 2020). Second-grade readers showed the same large post-training gains after HW as after kusho training and only for 4th-graders HW training was no longer as effective as kusho training (Xu et al., 2020). More important, when preliterate children (without pre-training knowledge) and first-grade beginning readers were trained on graphs (letter-like), only preliterate children showed larger post-training gains after HW than after a control training, whereas first-graders did not show any effect of type of training. More important, in this study, preliterate children showed even larger post-training gains after a (non-motor) training in mirror-image discrimination than after HW training (Williams, 1969; for a similar advantage from this visual-discrimination training over HW training see, Williams, 1975). This point is specifically about prior knowledge on the script to-be-learned and not about age (but see, Longcamp et al., 2005). Indeed, we known that, regardless of age of literacy acquisition, learning to read leads to the same benefit in orientation processing and mirror-image discrimination (e.g., Kolinsky et al., 2011; Fernandes et al., 2016) and visual graph recognition becomes underpinned by the vOT (e.g., Dehaene-Lambertz et al., 2018; Hervais-Adelman et al., 2019). The point here is that we cannot assume that evidence from participants with prior knowledge of the script can speak about the mechanisms involved when learning these visual graphs.

This observation also highlights a second loose end of this topic of research, which regards whether post-training gains in new (unknown) graphs by literate participants would generalize to those elicited in preliterate or illiterate participants (Naka and Naoi, 1995; Xu et al., 2020; Vinci-Booher and James, 2020; Vinci-Booher et al., 2021). This loose end is not specific to the stroke processing hypothesis, given that other accounts have also provided evidence with learners that had prior reading expertise in another script (Seyll et al., 2020; Vinci-Booher et al., 2021). However, this point is raised by this hypothesis because this is the only proposal that confuses evidence about visual graph recognition by fluent adult readers (i.e., with prior knowledge on these graphs) with evidence about learning of new (unknown) graphs (e.g., Parkinson and Khurana, 2007; Longcamp et al., 2016). More important, in training studies with adults (even if these studies adopted highly controlled, novel, artificial scripts; for a discussion on the advantages of artificial scripts, see, e.g., Chetail, 2017), HW on the first script was already automatic (Waterman et al., 2015; Julius et al., 2016; Palmis et al., 2020). In contrast, studies with preliterate children were conducted with learners for whom the HW training implied learning of the visual graphs plus learning of the HW task itself. The message here is that we do not know whether: (a) different cognitive mechanisms underpin the benefit from HW training by naïve participants (preliterate children or illiterate adults) and by readers (who are experts in letter and HW in a first script and for whom generalization might apply, if possible); or (b) the same mechanism is involved but the time course of the HW benefit might be modulated by reading expertise in another, first script. Surprisingly, although silent, the literature seems to have implicitly assumed the latter, given that studies with preliterate children have significantly longer training programs and on less graphs than those with literate adults (Araújo et al., 2021).

In fact, indirect evidence suggests that the mechanism is likely the same for learners that are either fully naïve or experts in another script; only the time course seems to differ. After phonological training (without HW), preliterate children and literate adults show similar enhancement in vOT response for the learned graphs (Brem et al., 2010, 2018). In what regards learning HW (in an invented-letter task), the same learning curve, the same improvement slope in consolidation (24 h post-training) and retention (follow-up after 2 weeks) was found in 5–6-year-old preliterate, 7–8-year-old beginning readers, and adults, although preliterate started with the lowest performance (Julius and Adi-Japha, 2015). When copying single letters, preliterate children took more time to complete the task, but both adults and children spent similar time inspecting the letter (in number and duration of fixations) before writing it down. Yet, children still inspected the visual item during writing, whereas adults showed a larger decrease in fixations (Maldarelli et al., 2015).

#### Stroke Processing Is Not About Stroke Order

Finally, it is becoming clear that evidence for a stroke effect is weak. In what regards, a stroke order effect, if there is motor simulation (re-instatement) during visual graph recognition, then presenting a consistent stroke order would prime perceptual end states (Parkinson and Khurana, 2007; Parkinson et al., 2010). Consequently, action-inconsistent sequences would interfere with visual graph recognition. It is not possible to predict facilitation in the former condition without predicting interference in the latter. This is the rationale of motor interference paradigms which are a credible evidence for a potential role of stroke processing in graph recognition by fluent readers (James and Gauthier, 2009).

Therefore, full examination of stroke order effects requires a proper baseline. Only then we can attest whether action-inconsistent sequences, which would activate incompatible graphic motor programs, and hence, incongruent graph representations, would interfere with visual recognition of a different graph (James and Gauthier, 2009). In fact, in studies with such baseline, action-inconsistent sequences did not interfere with visual graph recognition. For example, adult readers showed faster graph categorization when letters were primed by an inconsistent stroke order than by a static letter or a dynamic neutral circle (Parkinson and Khurana, 2007, Experiments 1 and 3). Thus, the inconsistent stroke order did not hinder graph recognition, it actually facilitated it, albeit less than the consistent stroke order. The same observation applies to the results of Patient NGN (Schubert et al., 2018) who showed an advantage in letter naming of dynamic letters. If such advantage was due to stroke order processing, then his letter recognition would have been hampered in the dynamic reversed (inconsistent stroke order) relative to the baseline condition. Instead, the inconsistent stroke order still led to better letter naming than the static condition. To be clear, both stroke orders led to better letter naming (accuracy: 89.4% for consistent stroke order; 80.3% for inconsistent stroke order; 73.7% for static letters) even though the inconsistent stroke order is unusual because it is contrary to the direction of writing (Simner, 1981). Notably, the results of Patient NGN even show that for 12 out of the 26 uppercase letters examined, the inconsistent order led to either better or as good performance as the consistent stroke order. If these results were about stroke (movement, dynamic) processing, then the inconsistent order would be compatible with a different letter, and hence, would interfere with visual graph recognition.

Likewise, when preliterate 5-year-old children were trained on new graphs *via* HW either with a self-defined or with a predefined stroke order, both groups showed similar post-training gains in graph recognition. More important, there was no advantage in visual recognition of dynamic graphs presented in the same (consistent) stroke order relative to a novel, inconsistent one (Merritt et al., 2020). The results by Wiley and Rapp (2021) also suggest that stroke processing is not the locus of the benefit from HW training. On the one hand, motoric representations derived from HW training differ from symbolic letter identities. On the other, dynamic training (mimicking stroke sequence) was not enough to lead to the emergence of abstract representations, given that all groups were exposed to dynamic graphs during training, but the group trained *via* typing did not show any hint of abstract letter representations after training (in contrast to what was found after training in HW or in visual categorization).

In sum, the overview of the literature presented in this second section shows that the stroke processing hypothesis is weak and sensorimotor representations are not able to explain the advantage from HW training when learning visual graphs. In fact, the available evidence, even the one that at first sight might seem compatible with sensorimotor representations can be accommodated by a perceptual learning mechanism. In the next section, we detail our proposal, integrating it with the available evidence. We also propose how to disentangle and to test the role of visual features and of strokes (motoric features) in future research. Table 1 presents a summary of the hypotheses derived and the predictions that follow from our perspective.

**TABLE 1:** Summary of our proposal.

**Putative mechanism**	**Symbolic representations involved**	**Predictions**	**Refuting evidence**	**Future directions**
**Stroke processing** (motoric primitives)	Abstract motoric	Dynamic letter presentation during training facilitates subsequent visual graph recognition	- Courrieu and de Falco, 1989;	Effects of dynamic graphs vs. static decomposed graphs
			- Wiley and Rapp, 2021.	
		Multi-system interplay, top–down effects from abstract motoric representations ⇒ late effects in training (in a later phase)	- Benefit of HW in very short, single session training (e.g., Naka and Naoi, 1995; Suggate et al., 2016);	- Manipulation of training regime: single vs. multi-session training (prediction: smaller benefit early on and for single-session training);
			- Larger benefit from HW training in naive participants (Williams, 1975; Xu et al., 2020).	- Potential involvement of sleep;
				- Time-course of HW training effects: EEG or eye-tracking methods.
		Larger benefit from HW training on highly confusable graphs (e.g., d and b) because of different motor representations ⇒ mirror-image letters (e.g., p and q) would lead to smaller interference than motoric similar letters (e.g., P and R).	(still untested)	Concurrent manipulation of visual vs. motoric similarity.
**Perceptual learning** (visual analysis)	Abstract graph identities	HW is a natural way of promoting detailed visual analysis and learning of diagnostic features, leading to emergence of abstract graph identities ⇒ visual analysis and HW trainings result from operation of the same mechanism.	None (all studies where visual analysis vs. HW trainings show the same magnitude of benefit in graph recognition (e.g., Williams, 1969; Seyll et al., 2020).	- Bayesian statistics;
				- Multivariate pattern analysis of brain responses with EEG;
				- Training naïve vs. literate participants;
				- Manipulation of script proximity (e.g., diagnostic features, writing direction);
				- Generalization effects to untrained graphs.
		Larger benefit from HW training on highly confusable graphs (e.g., d and b) because of visual segmental analysis ⇒ mirror-image letters (e.g., p and q) would lead to larger interference than motoric similar letters (e.g., P and R)	(Still untested)	Concurrent manipulation of visual vs. motoric similarity.

## Our Proposal: Disentangling Strokes From Visual Features

Stroke representations are not necessarily motor, gestural in format. In fact, sensorimotor representations are already a transduction (Machery, 2016). The emphasis by Rapp and colleagues (e.g., Rapp and Caramazza, 1997; Dufor and Rapp, 2013; Rothlein and Rapp, 2014) in the abstract, amodal quality of these representations highlights their symbolic nature, which gives them computational fitness (Mahon and Hickok, 2016). Indeed, the PMd, sometimes referred as a motor center (Roux et al., 2009; Longcamp et al., 2016), is involved in serial sublexical orthographic processes shared by writing and by pseudoword reading (Pattamadilok et al., 2016). From the reviewed evidence, it is clear that the benefit from HW is not about the motor act itself (e.g., Courrieu and de Falco, 1989; Xu et al., 2013, 2020). Our proposal is that the benefit from HW training when learning visual graphs is about computations over symbolic representations (for a similar proposal and supporting empirical evidence, see Wiley and Rapp, 2021). Whether these regard strokes (units of movement, primitives of HW), visual features (image components, primitives of visual object recognition), or both must first be seriously discussed. Several authors have pointed out that visual analysis and dynamic movement could be involved (e.g., Courrieu and de Falco, 1989; Cao et al., 2013a; Merritt et al., 2020; Vinci-Booher and James, 2020), but few studies tried to disentangle them.

### What If Stroke Processing Were Involved in the Benefit From Handwriting Training?

We propose that if stroke processing were a core mechanism, then it would be about multi-system interplay, where top–down information from motor (and also possibly from phonological) system (Pegado et al., 2014) would assist subsequent graph recognition due to automatic spreading of activation within the orthographic network. If the benefit from HW is about top-down processes, then it would take time to evolve and, even when established, it would occur at a later stage in processing.

Indeed, Vinci-Booher and James (2020) have suggested that an extensive amount of experience may be required for parietal-frontal regions to develop a functional response during letter perception. Such top–down effects could also explain why the benefit from HW training is sometimes labile (that is, not always observed even in well-controlled studies: e.g., Naka and Naoi, 1995; Kiefer et al., 2015). Note, however that such flexible expression already suggests that stroke processing might not be a core operation. This mechanism is also incompatible with the observation of a benefit from HW training after a single training session of less than 20 min by preliterate children (e.g., Li and James, 2016; Guan and Wang, 2017) and of larger gains in post-training graph recognition in learners who are naïve to graphs or have less reading experience (Williams, 1969; Xu et al., 2020). Regarding the temporal course of a putative top–down effect of stroke processing during visual graph recognition, such effect would occur at a later time-window, whose assessment implies the adoption of high-temporal resolution methods as eye movement recordings or electroencephalography (EEG). However, to our knowledge, no study has hitherto examined this hypothesis.

Note that such stroke processing would be especially relevant for highly confusable graphs like mirror images (e.g., d and b), whose discrimination would be facilitated by bidirectional connections between abstract graph representations and the different motoric representations (Pegado et al., 2014; Longcamp et al., 2016). This prediction is compatible with the available evidence. However, it is also compatible with the operation of a perceptual learning mechanism (for a discussion, see also, Araújo et al., 2021).

### Perceptual Learning and Visual Segmental Analysis Might Be a Parsimonious Account

Given the inconsistent evidence, we propose that rather than about stroke processing, another mechanism could be responsible for the benefit from HW training. From the evidence reviewed thus far both the stroke processing hypothesis and the perceptual variability hypothesis are limited. However, the visual analysis hypothesis is promising, given that none of the available evidence is incompatible with it. The major problem of this hypothesis is that few studies have systematically examined it, while testing the alternatives. We join other authors (e.g., Gibson, 1970; Williams, 1975; Kaufman, 1980; Courrieu and de Falco, 1989; Mayer et al., 2020; Seyll et al., 2020) and in what regards the benefit from HW training when learning visual graphs, we propose that HW is a vehicle for optimizing perceptual learning of the new visual graphs.

Letter recognition involves perceptual processes based on the extraction of elementary visual features (Pelli et al., 2003, 2006). Letter knowledge, the gateway for reading across reading development (Grainger, 2018), comprises knowledge of the letter form and of phonological correspondences. Letter discrimination is a very low-order aspect of reading, but it precedes decoding graphs into phonological counterparts: “the discovery of distinguishing characteristics and the extraction of invariant orders, both set up as relational observations, are the ultimate prerequisites for learning to read. It is only after such discriminations are learned that recognition and production (writing) can be achieved” (Kaufman, 1980, p. 57).

The visual system is highly solicited during HW, and hence, HW is an optimal vehicle for perceptual learning. To become able to successfully reproduce a graph, HW must rely on a detailed and explicit visual analysis of the graph, especially of diagnostic features, and their relationship in shape, orientation, and visuospatial arrangement (Courrieu and de Falco, 1989; Seyll et al., 2020). One must learn the features that are critical to become able to differentiate visual graphs (Gibson et al., 1962; Gibson, 1969, 1970). This is all about perceptual learning, which establishes a deep relationship between perception and experience (Gilbert and Li, 2012; Goldstone and Byrge, 2015). Furthermore, this proposal agrees with evidence on eye movement patterns during copy of single letters by preliterate children and literate adults (Maldarelli et al., 2015).

We are not arguing that motor learning is not part of the learning experience promoted by HW, but rather that the benefit from HW training when learning visual graphs is especially because HW is a natural way of promoting detailed visual analysis (Seyll et al., 2020). The rationale is that the benefit from HW is because this training implies visual analysis, facilitating the creation of perceptual representations that then underpin visual graph recognition. Therefore, even when training is non-motor, without a graphomotor task, if it taps into graphs’ diagnostic features, then the gains will be as large as the benefit from HW training.

The available evidence, since the earliest studies until the most recent ones (e.g., Williams, 1969; Seyll et al., 2020), fully agrees with this prediction. When training implies visual discrimination of distinctive features of graphs, either *via visual composition* (where participants are presented with several individual features and select those that compose the graph: Seyll et al., 2020), *segmental, non-dynamic* (where the presented graph is decomposed into its static features: Courrieu and de Falco, 1989), *match-to-sample* (where learners select from a set, including mirror images and plane rotations, which one corresponded to the graph, with feedback on response: Williams, 1969, 1975), the gains in visual graph recognition are similar (or even larger: Williams, 1969, 1975) to those after HW training. To the best of our knowledge, no study showed significantly larger benefits from HW training when compared to non-motor visual segmental conditions requiring explicit visual analysis or attending to distinctive features of graphs. Furthermore, all the available evidence on which HW training led to the larger benefit in subsequent visual graph recognition can be accommodated by a visual analysis account.

Notably, perceptual learning and visual analysis can also accommodate evidence that at first sight seems compatible with a stroke processing’s explanation (Parkinson and Khurana, 2007; James and Gauthier, 2009; Parkinson et al., 2010; Schubert et al., 2018). Not only the stroke processing hypothesis has serious limitations (as discussed in section “The stroke processing hypothesis”) but, more important, for all studies in which visual feature and stroke processing were not disentangled, either one could be the key factor because many graphs are similar both in visual features and in strokes. Indeed, most studies suffer from this fundamental ambiguity regarding similarity (Rapp and Caramazza, 1997). For studies that did not find stroke interference relative to the static baseline (e.g., Parkinson and Khurana, 2007; Schubert et al., 2018), the visual analysis’ explanation is credible. Regardless of stroke order being consistent or not, the presentation of decomposed visual items (e.g., in Parkinson and Khurana, 2007; Parkinson et al., 2010; Itaguchi et al., 2015; Schubert et al., 2018) necessarily implies presentation of separate visual features, enhancing visual analysis of graphs, as happens in HW training (e.g., Williams, 1969; Courrieu and de Falco, 1989; Seyll et al., 2020).

Mere visual exposure to highly confusable graphs is not enough. When trained on mirror-image graphs, learners who were trained on graphs’ shape only (not on the diagnostic feature) showed worse subsequent visual graph recognition than learners whose training focused on orientation (the diagnostic feature; Pick, 1965; Caldwell and Hall, 1969, Experiment 1; Williams, 1969; Tawney, 1972; Samuels, 1973; Spectorman et al., 1977). More interesting, these pioneer studies showed that such focus in diagnostic features leads to *generalization* from the trained graphs to novel, untrained ones, including real letters (Pick, 1965; Tawney, 1972; Nelson and Wein, 1974). Non-motor visual training can thus boost mirror-image discrimination. This is not a paradox because mirror-image invariance is a perceptual bias (Bornstein et al., 1978; Kolinsky et al., 2011). It is not a low-level visual property computed in early visual occipital areas (Dehaene et al., 2005). Indeed, mirror images (e.g., d and b) have very different retinal projections. Their perceptual equivalence occurs at a higher level (like that of structural description of non-linguistic objects), underpinned by the vOT (Logothetis et al., 1995; Dehaene et al., 2015).

More important, visual analysis promoted by HW could also explain the supposed motor interference by letters, given that the two properties were intertwined (James and Gauthier, 2009). We all agree that graphs are not just visual objects, but graph shapes, graph names, and graph motor plans are representational dimensions that are dissociable (Rothlein and Rapp, 2014; Zhai and Fischer-Baum, 2019; Wiley and Rapp, 2021). Therefore, to test our alternative, one must manipulate the similarity of concurrent letters within-trial in terms of visual features and in terms of strokes. If HW assists in visual segmentation, then greater interference would be found for letter pairs that share more visual features, regardless of their (dis)similarity in strokes. If it is about activation of motoric representations, then pairs composed of highly confusable letters as mirror images (e.g., p and q), which share visual features except orientation but do differ in motor strokes, would elicit significantly less interference than letters that are visually less similar but closer in stroke composition (e.g., P – R). The acid test is one in which visual feature processing and stroke processing are confronted (see Table 1).

Although this strategy has been rare, such studies are particularly revealing (Courrieu and de Falco, 1989; Zhai and Fischer-Baum, 2019; see also Rapp and Caramazza, 1997). Indeed, Zhai and Fischer-Baum (2019) showed that visual similarity of graphs was the best predictor of kanji recognition (in a same-different matching task) for adults who were either readers of Chinese or not (the latter were Latin-alphabet readers of English). It was only for Chinese readers that phonology and semantics also tended to be significant predictors. Stroke processing was never a reliable predictor of kanji discrimination. In fact, even when stroke similarity was the only predictor considered (and even when it included as parameters: sequence of component strokes, shared first stroke, stroke bigram familiarity, stroke-motor features), it was still not a reliable predictor. Bayesian statistics further demonstrated that stroke similarity had no contribution at all for kanji discrimination either by naïve or expert observers (Zhai and Fischer-Baum, 2019). However, these results speak to the mature reading system and not to the benefit that HW training could have when learning visual graphs.

In this regard, the results of the training study by Courrieu and de Falco (1989) with 3–6 years old preliterate children are especially revealing. Relative to a control visual-only group (non-segmental non-dynamic), the group trained on letters *via* HW showed similar post-training gains in visual letter recognition as the groups trained on letters presented broken down into static visual features either without HW (segmental non-dynamic group) or with HW training (segmental dynamic group). There was no added value of HW; the key factor was visual analysis *via* segmental training and not stroke processing. These results also highlight another aspect that deserves to be examined in future studies, which regards whether dynamic stimuli without HW (which has been used in some research to mimic stroke order without a motor action; e.g., Parkinson and Khurana, 2007; Schubert et al., 2018; Merritt et al., 2020) could fully elicit visual segmental analysis. To our knowledge no study has yet compared these two training conditions, that is, dynamic unfolding vs. static visual decomposition of graphs. However, the results of Wiley and Rapp (2021) suggest that dynamic unfolding is not enough given that when learners were exposed to dynamic letters, training *via* typewriting did not lead to the emergence of symbolic graph representations. It was only training *via* visual categorization of graphs (graph/non-graph decision) or *via* HW which led to the emergence of symbolic representations. Note that the visual (active) training in Wiley and Rapp (2021) did not focus on either decomposed or diagnostic features of graphs; it just involved symbol/non-symbol categorization. Indeed, pioneer research has clearly shown that visual training only leads to the same benefit as HW training when the former is fully focused on diagnostic features like orientation (e.g., d and b; Koenigsberg, 1973; Williams, 1975) or when visually segmented graphs are presented during training (Courrieu and de Falco, 1989). Mere visual exposure is not enough to elicit visual segmental analysis (e.g., Pick, 1965; Caldwell and Hall, 1969, Experiment 1; Williams, 1969; Tawney, 1972; Samuels, 1973; Spectorman et al., 1977).

## Discussion

Cognitive science has recently shown a renewed interest in the role of HW training when learning to read (for an overview, see, James, 2017). Indeed, a large body of empirical evidence supports the advantage from HW training relative to control training in subsequent visual graph recognition (Araújo et al., 2021; for an overview, see, James, 2017; e.g., Williams, 1969; Longcamp et al., 2006, 2008; James, 2010; Bara and Gentaz, 2011; Guan et al., 2011; Suggate et al., 2016; Labat et al., 2020; Mayer et al., 2020; Seyll et al., 2020). However, the nature of the underlying cognitive mechanism has been elusive and rarely addressed (Gibson, 1970; Williams, 1975; Kaufman, 1980; Li and James, 2016; Zhai and Fischer-Baum, 2019; Mayer et al., 2020; Seyll et al., 2020; Vinci-Booher et al., 2021). The available theoretical proposals have hitherto been unspecified. Therefore, it was not fully clear which predictions would follow and which patterns of performance would empirically distinguish them. In this work, we presented the most promising theoretical accounts, detailed their predictions, and critically revisited key empirical evidence.

We join other authors (e.g., Gibson, 1970; Kaufman, 1980; Courrieu and de Falco, 1989; Mayer et al., 2020; Seyll et al., 2020) in the proposal that HW training is a vehicle for perceptual learning of visual graphs. Visual segmental analysis would be the key element in HW training by highlighting diagnostic features of visual graphs which then would assist the emergence of perceptual representations to be involved in subsequent visual graph recognition (see also Wiley and Rapp, 2021).

Although beyond the scope of the present work, the theoretical proposals discussed here have implications for the nature of mental representations. Embodied and symbolic cognitive accounts are two perspectives with dramatically different approaches in this regard. Note that both proposals are able to accommodate the available fMRI evidence (e.g., Longcamp et al., 2003, 2008; James and Atwood, 2009; James, 2010; James and Engelhardt, 2012; Nakamura et al., 2012; Vinci-Booher et al., 2016, 2021; Vinci-Booher and James, 2020) but they do differ on the cognitive mechanisms responsible for such effects. According to the embodied sensorimotor accounts, the fronto/parietal regions within the writing network are activated when viewing graphs or written words because these regions underpin motoric representations that are core of orthographic (sensorimotor) representations (e.g., Parkinson and Khurana, 2007; Longcamp et al., 2008, 2016; James and Atwood, 2009; Cao et al., 2013b; Itaguchi et al., 2015). According to symbolic accounts, the observed motor activation is rather due to information spreading throughout the orthographic system; it is coactivation, not causation (e.g., Rapp and Caramazza, 1997; Rothlein and Rapp, 2014; Mahon and Hickok, 2016). In fact, from the reviewed literature, we must conclude that there is no compelling evidence that embodied representations are necessary for understanding the benefit from HW training in visual graph recognition (for a meta-analysis and discussion, see, Araújo et al., 2021). In fact, the recent results of Wiley and Rapp (2021) show that HW training leads not only to the emergence of motoric representations but also of dissociable symbolic orthographic representations.

The present work has also raised several questions to be addressed in future research. We thus present further predictions and future directions that could be enlightening in what regards the mechanism underpinning the benefit from HW training when learning visual graphs. First, although the idea of a perceptual learning mechanism *via* visual analysis seems parsimonious, it is mainly corroborated by behavioral evidence showing that this type of visual training leads to either similar or even larger post-training gains than HW training (e.g., Williams, 1969, 1975; Koenigsberg, 1973; Courrieu and de Falco, 1989; Li and James, 2016; Seyll et al., 2020). In the present work, we discussed how this visual analysis hypothesis could explain prior evidence which did not test it nor considered it. We also presented the arguments in favor of this proposal, considering detailed predictions. However, we must acknowledge that even if the behavioral effects are the same, the benefit from visual segmental analysis and HW trainings might result from different neurocognitive mechanisms. Cognitive, neural, and behavioral changes when learning visual graphs will be closed intertwined and all are relevant for understanding the underlying mechanism. At the cognitive level, we believe that the predictions presented here, especially those regarding the opposition between stroke and visual feature processing, will be especially revealing (see Table 1). In what regards the neural implementation, a promising technique to address the underlying mechanism is *multivariate pattern analysis* of brain responses, especially when adopted with high-temporal resolution techniques as EEG (King and Dehaene, 2014). Such classification algorithms could assist in accomplishing three aims: (i) determining the temporal course of emerging representations of graphs in the brain; (ii) testing whether the brain pattern of response is able to predict post-training gains in visual graph recognition; (iii) testing whether one can predict which type of training the participants were in (e.g., HW vs. visual analysis) based on patterns of brain responses to visual graphs after training. Achievement of these aims would be especially revealing on whether the similar benefits from HW training and visual segmental training (e.g., Courrieu and de Falco, 1989; Seyll et al., 2020) do reflect the operation of the same neurocognitive mechanism and on whether top–down effects related with a late stage of processing would be involved in the benefit from HW training (see Table 1). In this regard, convergence across methods will provide a better characterization of the components involved. This line of research is thus relevant to the future development of our framework.

Second, we also discussed two loose ends of this topic that hopefully will be considered in future research. We are hardly the first to consider them, for which the earliest studies contributed with deep insights (e.g., Tawney, 1972; Williams, 1975; Kaufman, 1980; Kirk, 1980), albeit these pioneer works have (surprisingly) been underestimated by recent research. On the one hand, *training regime* (i.e., total amount, frequency, and duration of training) has been overlooked (although with references by Jeffrey, 1958; Longcamp et al., 2006), probably because the benefit from HW has been found both in single and multiple session training (e.g., Williams, 1969; Li and James, 2016; Mayer et al., 2020; Seyll and Content, 2020). However, the nature of graph representations may change as a function of training regime, which would agree with the different patterns of fMRI activation found when children (6-year-old beginning readers and 8-year old) and adults (fluent readers) viewed letters (Vinci-Booher and James, 2020) and the observation that functional connectivity between visual and motor brain regions found immediately after HW training were already gone after one week (Vinci-Booher et al., 2021).

Notably, a manipulation of training regime could also be key in hypothesis testing. If the benefit from HW training were about stroke processing, it would take time to develop, and hence, the benefit relative to control training would increase along sessions. Alternatively, if the benefit from HW training is due to visual analysis, then the largest difference relative to control training would occur early on, which would dissipate with stabilization of graph representations. Nonetheless, whenever testing of visual graph recognition occurs multiple times along training, then the contribution of testing for the learning curve must be controlled to ensure that it is not confounded with the independent contribution of training (for this kind of strategy, see, Wiley and Rapp, 2021). Training along multiple sessions also allows for the involvement of sleep, which is relevant in perceptual learning and visual discrimination (Stickgold et al., 2000). Indeed, sleep has a significant role in the stability of post-training gains in mirror-image discrimination of graphs after HW training (Torres et al., 2020). In this vein, follow-up assessment is of critical importance (Longcamp et al., 2005, 2008; Vinci-Booher and James, 2020; Vinci-Booher et al., 2021). However, very few studies included a follow-up and with a disperse interval, from one week to several months. When the new graphs were real letters, these studies also had the possible confound of uncontrolled post-training exposure (e.g., Longcamp et al., 2005).

On the other hand, when learners are already experts in reading and HW in their first script, a literacy-specific network is already established. Therefore, they might rely on it when learning new graphs. The research has suggested, however, that this might not be the case. Indeed, neurocognitive and behavioral effects of leaning a new script are similar in (literate) adults and (preliterate) children, even though besides reading skills there is also an age/maturation confound in this comparison (James and Atwood, 2009; Brem et al., 2010; James, 2010; James and Engelhardt, 2012; Brem et al., 2018; Vinci-Booher et al., 2021). Additionally, the earliest studies have examined possible generalization effects from the graphs trained *via* HW to novel, untrained graphs, and found that such generalization occurred and assisted subsequent learning of letter-sound correspondences (Pick, 1965; Tawney, 1972; Nelson and Wein, 1974). However, since then this generalization effect has not been considered, leading to several questions. For example, could generalization effects depend on the type of diagnostic features of the new script and whether these features resemble or not those of the first script? And if this were the case, with which time course?

These questions also highlight the link between the studies on the benefit from HW training when learning visual graphs and the field of perceptual expertise. Indeed, perceptual expertise can be contrasted to other forms of perceptual learning as it is characterized by robustness and generalization to new contexts and to new items within the expertise domain (Curby and Gauthier, 2010). This field has systematically focused on the interaction between participant (naïve vs. expert), item (novel vs. old), and task demands (individuation vs. categorization), which are all known to be important in development and in expression of expertise.

In fact, the benefit from HW training when learning visual graphs is, in our view, a paradigmatic example of perceptual and of motor expertise. Note that, in literate adults, HW is highly automatic; it consists of rapid sequences of short movements with fast changes in direction, resulting in high-quality, stable, and consistent reproduction of graphs. It is clearly an ecological example of motor expertise like playing a musical instrument (Palmis et al., 2017; Calmels, 2020). Therefore, the convergence with research from perceptual and motor expertise could be fruitful for a deeper understanding of the cognitive mechanism underpinning the benefit from HW training in visual graph recognition (see, e.g., Folstein and Monfared, 2019). This research is also relevant for the expertise literature, given that it is easier to study expertise in a domain with many participants and where relevant stimuli is widely available.

In summary, the investigation of the cognitive mechanism underpinning the benefit from HW training when learning visual graphs goes beyond its realm. It can provide insights into the principles, limits, and possibilities of learning of cultural activities. It is also relevant for generation of testable hypotheses about interactions between training modes and performance benefits. In a broader scope, it also speaks to the nature of cognitive representations. Finally, it has relevance for Education and public policy because it can translate in better designing of literacy programs. When learning visual graphs, HW involves multiple components. Thus, we can no longer advocate the adoption of HW as a holistic school activity. The critical processes can and should be separated in order to implement the best educational strategies in literacy instruction.

## Data Availability Statement

Publicly available datasets were analyzed in this study. This data can be found here: OSF Preprints. doi: 10.31219/osf.io/quywt; https://osf.io/wzdxn/.

## Author Contributions

TF and SA conceptualized together this work, critically revised the manuscript, and prepared the revised version. TF has the first authorship and wrote the first draft of the manuscript. Both authors approved the final version submitted and the revised version.

## Conflict of Interest

The authors declare that the research was conducted in the absence of any commercial or financial relationships that could be construed as a potential conflict of interest.

## Publisher’s Note

All claims expressed in this article are solely those of the authors and do not necessarily represent those of their affiliated organizations, or those of the publisher, the editors and the reviewers. Any product that may be evaluated in this article, or claim that may be made by its manufacturer, is not guaranteed or endorsed by the publisher.

## Footnotes

1 The term graph is preferable to letter because it applies to orthographic characters of any written system and not just to alphabets (cf. Chang et al., 2018). In the present work we also adopt the term *visual graph* when referring to the visual component of these orthographic units given that graphs (e.g., letters) have multiple facets (i.e., visual, phonological, motor).
